# ‘When they come, we don’t send them back’: counter-narratives of ‘medical xenophobia’ in South Africa’s public health care system

**DOI:** 10.1057/s41599-019-0309-7

**Published:** 2019-09-03

**Authors:** Kudakwashe P. Vanyoro

**Affiliations:** 1 African Centre for Migration & Society, University of the Witwatersrand, Johannesburg 2000, South Africa

## Abstract

Relying on the experiences of migrant patients, research on migration and health in South Africa has documented a particular concern with public health care providers as indiscriminately practicing ‘medical xenophobia’. This article argues that there is more complexity, ambivalence, and a range of possible experiences of non-nationals in South Africa’s public health care system than the current extant literature on ‘medical xenophobia’ has suggested. Based upon in-depth interviews with frontline health care providers and participant observation at a public health care clinic in Musina sub-District, this article demonstrates how discretion may play a crucial role in inclusive health care delivery to migrants in a country marred by high xenophobic sentiment. It finds that in spite of several institutional and policy-related challenges, frontline health care providers in Musina provided public health care services and HIV treatment to black African migrants who are often at the receiving end of xenophobic sentiment and violence. The article concludes that citizenship, nationality or legal status alone do not appear to tell us much as ‘bureaucratic incorporation’ and ‘therapeutic citizenship’ are some of the modalities through which migrants are constantly being (re)defined by some of South Africa’s health care providers.

## Introduction

The fieldwork for this article began with the expectation that the investigation focusing on health care providers would reveal their practices to be exclusionary and xenophobic. On the first day of fieldwork, Dorcas, the Facility Manager at the clinic in Musina glanced at the long queue of patients and said, ‘You’ll never know where they are from until you start treating them’. She appeared to be suggesting that the community here was diverse, in terms of nationality, culture and language. Located in a local settlement that was approximately 12 km from the Beitbridge border between Zimbabwe and South Africa, local inhabitants mostly spoke in Venda, Shangaan, Tsonga, Sotho, while most Zimbabwean migrants spoke either Shona or Ndebele. While Dorcas and her staff were overloaded and understaffed in this context of high mobility, Dorcas maintained that, ‘we don’t let a patient die because they have no ID. We are here to save lives’.

As the study later found, in spite of several institutional challenges, frontline health care providers in Musina provided public health care services and HIV treatment to black African migrants who are often at the receiving end of xenophobia in the country. These frontline health care providers bypassed institutional and policy-related difficulties related to registering and treating three categories of ‘indigent’ migrant patients: undocumented migrants, non-native speaking migrants and migrants without referral letters (‘self referrals’).

Hence, this article argues that there is more complexity, ambivalence and a range of possible experiences of non-nationals in South Africa’s public health care system than the current extant literature on ‘medical xenophobia’ has suggested. Adhering to Chimamanda Ngozi Adichie’s trope of ‘the danger of a single story’ that tends to create a critical misunderstanding, the article shows that frontline health care providers adopt a counter-intuitive response; the stereotyping of migrant patients to blame them for their ‘indigency’, which is simultaneously accompanied by innovation and creativity to assist them through various rationales (Lipsky, 2010). Altogether, this multifaceted response culminates into ‘bureaucratic incorporation’ (Marrow, 2012; Gell-Redman et al., 2014; Walter and Schillinger, 2004) and to some extent emerging forms of ‘therapeutic citizenship’ (Nguyen et al., 2007; Wilhelm-Solomon, 2013; Young et al., 2019).

It has been established that existing policy responses to communicable diseases in South Africa and the southern African region do not adequately engage with mobility (Southern African Development Community (SADC), 2009; Walls et al., 2015; Vearey et al., 2016; 2017). Treatment guidelines in South Africa have been found to either be incomplete or inapplicable to migrant patients and SADC policies and programs to deal with communicable diseases such as the Human Immunodeficiency Virus (HIV) in practice do not extend to migrant patients (Walls et al., 2015; Vearey et al., 2016; 2017). This scenario makes frontline discretion unavoidable as health care providers have to rely on their own judgment to determine what ‘best practices’ to invoke. Their discretion lies in the ability to make decisions about how they will administer policies and programs with relatively little input or interference from other institutions (Birkland, 2015, p. 119).

While policy-makers and planners are recognizing that migration is both a key social determinant of health (MacPherson and Gushulak, 2001) and structural driver of HIV (Zuma et al., 2003; Abdool Karim et al., 2011), migration in South (ern) Africa is rarely well managed and tends to be precarious, clandestine and irregular, as responses to migration are inadequate (Fonn, 2011; Walls et al., 2015; Vearey et al., 2016). It has been found that when migrants move, they not only move across geographical borders, ‘but also across, between, and among medical systems’ (Sargent and Larchanché, 2011, p. 346). Yet, there is a gap in clear policy, standardized guidelines and systems of patient referral to harmonize and better coordinate HIV treatment in the SADC region (Walls et al., 2015; SADC, 2009; Vearey et al., 2017).

Relying on the experiences of migrant patients, research on migration and health in South Africa has documented a particular concern with public health care providers as indiscriminately practicing ‘medical xenophobia’ (Zihindula et al., 2015; Crush and Tawodzera, 2014; Hunter-Adams and Rother, 2017; Misago et al., 2010; Human Rights Watch, 2009; Landau, 2007; Pursell, 2007; Nkosi, 2014). According to the South Africa National Health Act, primary health care facilities, which consist of clinics and community health centers, must provide free care to everyone, except for people covered by medical aid schemes, or receiving payment from the workers’ Compensation Fund. Refugees are indeed entitled to the same access to treatment and ‘basic health care services’ as citizens in public health care facilities (Section 27 (g) of the 1998 Refugees Act). This also applies for undocumented migrants who are citizens of any SADC country.

In this context, when migrants are denied access to any form of treatment, the term ‘medical xenophobia’ is often deployed by scholars in order to describe the negative attitudes and practices of health sector professionals and employees towards refugees and migrants (Crush and Tawodzera, 2014; Zihindula et al., 2015). Zihindula et al. (2015: p. 2) define medical xenophobia as any practice, judgment or behavior that creates and strengthens oppressive relations or conditions that marginalize, exclude and/or confine the lives of refugees. Crush and Tawodzera describe medical xenophobia as ‘the negative attitudes of health sector professionals and employees towards refugees and migrants on the job’ (Crush and Tawodzera, 2011). The lack of specific reference to health and focus on attitudes and not health care delivery in these definitions reflects a particular generalization that also exists in public health discourses about how health providers are perceived to treat African migrants in South Africa.

Certainly, the question of a generally struggling health system is not sufficiently considered by migration and health studies that arrive at the former position. For example, even though many migrants seeking health care in South Africa’s public health care system face challenges arising from being ‘foreigners’ and indeed some challenges are more specific to them (Human Rights Watch, 2009), it has been found that South Africans can also face challenges within the public health care system (Vearey, 2012; Vearey et al. 2016; Vearey, 2008; Vearey, 2014). These challenges are related to the general shortages of medical personnel through attrition of staff to the private sector and emigration that hamper South Africa’s public health care system (Jobson, 2015; Segatti, 2014, p. 12). Other challenges that have been noted include high bed occupancy, high workload, low morale among nurses in public health care facilities and a burden of the HIV pandemic with 11.2% of the population of 54.9 million living with the disease (Jobson, 2015; Harrison, 2010).

In view of these often neglected systemic issues, other studies do not directly support the concept of medical xenophobia (Vearey et al., 2016; Pophiwa, 2010; Makandwa, 2014). In addition to these studies, this article provides a descriptive account of the various practices adopted by frontline health care providers to navigate systemic challenges and ‘blurred’ migration and health policies in ways that facilitate migrants’ access to health care; and more specifically access to antiretroviral treatment (ART). By exploring the wide range of innovations that frontline health care providers employ to address systemic and structural challenges presented by ‘blurred’ policies, through in-depth interviews and participant observation in a clinic located in Musina sub-District, this article demonstrates how discretion plays a crucial role in inclusive health care delivery to migrants in a country marred by high xenophobic sentiment.

This article’s focus on health care providers responds to existing calls for migration and health studies that move beyond methodological nationalism: ‘the assumption that the nation/state/society is the natural social and political form of the modern world’ therefore different national groupings ought to be studied individually (Myroniuk and Vearey, 2014). This approach has been criticized for assumming an ‘apparent naturalness and givenness of a world divided into societies along the lines of nation-states’ thus the analysis that follows from it does not rely on explicit reference to larger social entities (Myroniuk and Vearey, 2014; Wimmer and Schiller, 2003).

Studies of migration and access to social services have indeed been criticized for falling into ‘methodological nationalism’ in terms of how migrants are defined and conceptualized. While some studies have started to grapple with this bias, migrant exceptionalism—focusing exclusively on an individual non-national sample—has led scholars interested in access to health care services for migrants in South Africa to indiscriminately focus on the narratives of the migrants, with little attempt to understand the perspectives of local health care providers (and citizens) for a balanced and measured empirical critique. While newer studies have grappled with the fact that neither group can be studied without the other (Vearey et al., 2016), most scholars who have studied access to health care for migrants have generalized their findings to different South African contexts without adequately considering the heterogenity of South African service providers’ perspectives, experiences and practices and how these are affected and influenced by different structures and spaces. This article concludes that citizenship, nationality or legal status alone do not appear to tell us much about anything as bureaucratic incorporation and therapeutic citizenship are some of the modalities through which migrants are being (re)defined by some of South Africa’s health care providers.

The article is divided into five sections. It starts by situating the discussion of migration and health within an overview of migration, public health, xenophobia and medical xenophobia in South Africa. In this section, the article highlights the context of migration and xenophobia and identifies some of the theoretical and practical strengths and limits of the notion of medical xenophobia. In the second section, the article proceeds to situate the discussion of medical xenophobia within existing theories of bureaucratic incorporation and therapeutic citizenship. It briefly traces the role of both modalities to processes of inclusion. In the third section of the article, the methodology is presented. Fourth, the article considers the ways through which the practices of frontline health care providers in Musina challenge medical xenophobia by demonstrating the complexity, ambivalence and range of possible experiences of migrants in South Africa’s public health care system. Last, the article offers a discussion and some final concluding thoughts.

### Migration, public health, and xenophobia in South Africa

Southern Africa is characterized by mixed migration flows and is composed of people moving to seek livelihoods and as a result of forced migration (Landau and Segatti, 2009; Vearey, 2012; International Organisation for Migration (IOM, 2013). Declining economies, armed conflict and political uncertainty have contributed to migration becoming a strategy for people to safeguard themselves and their families, improving their livelihoods and safety in the process (Conway and Cohen, 1998; McCarthy et al., 2009; Kankonde, 2010). Migration is now a way of life for many. An estimated 3.3% of the world’s population has crossed a border (Weine and Kashuba, 2012; United Nations Population Fund (UNFPA, 2015).

Reflecting these global trends, an estimated 3.3% of South Africa’s population was born outside of the country (Statistics South Africa, 2015). Results from 2011 South Africa Census data analysis revealed that there were 2,173,409 international migrants (4.2% of the 2011 total population) (Statistics South Africa, 2014). Latest IOM figures suggest that South Africa is the most significant destination country in Africa, with around 3.1 million international migrants residing in the country (or around 6% of its total population) (IOM, 2017). Standing at 7% of the total South African population (Moultrie et al., 2016), internal migration is far more significant than international migration (Polzer Ngwato, 2010; Crush, 2011; Statistics South Africa, 2011).

Health is a key asset for all these migrants as they have been found to be often individuated through ‘positive selection’. Positive selection is the disposition of the healthier, younger population to move since the majority of migrants move in search of livelihoods, which requires an optimal degree of physical health. Also, arriving migrants are often healthier than the local population in what is otherwise known as the ‘healthy migrant effect’ (Vearey, 2014; Sargent and Larchanché, 2011; Wingate and Alexander, 2006).

In this context, xenophobia remains a widespread and growing concern (Harris, 2001; Black et al., 2006; Crush, 2008; Neocosmos, 2008; Crush and Frayne, 2010; Landau, 2011; Misago, 2011). Xenophobia and intolerance are indeed a recurrent reality in South African politics (Thakur, 2010). Foreign nationals have been attacked repeatedly in South Africa since 1994 (Adjai and Lazaridis, 2013). According to Neocosmos (2008), South Africa has experienced a ‘massive’ problem of xenophobia since 1994. This xenophobia is mainly targeted at African migrants, with some categories such as Nigerians being more criminalized in popular discourses than others (Neocosmos, 2008). Klotz (2016) argues that ‘the conflation of xenophobia with racism does not capture the South African situation, where anti-foreigner sentiments among Africans primarily target other Africans’. Nonetheless, xenophobia in South Africa is a form of discrimination closely related to racism because foreign status is declared on the basis of the crudest racist stereotypes (Neocosmos, 2008). This analogy is however not meant to undermine racism, because its attributes, ideologies and characteristics are completely different altogether; only to show that xenophobia is most likely to affect anyone or any group that is considered non-indigenous or non-autochthonous.

Xenophobic attacks continue to recur as ‘indigenous’ South Africans target perceived ‘foreigners’ whom they blame for their social and economic problems or ‘stealing the fruits of democratization’ (Klotz, 2016, p. 180). It is well known that today South Africa remains one of the most unequal societies in the world. The shift in political power has brought about a range of new discriminatory practices and victims and the foreigner is one such victim. Foreign migrants are constant targets and victims of xenophobic attacks. Xenophobia manifests itself as a spillover of citizen opposition to migration and a by-product of political scapegoating which blames migrants for the country’s unemployment woes. It has been observed that high expectations for employment, housing and other social provisions, coupled with the realization that delivery of these is not immediate, result in frustration targeted at foreigners (Lerner et al., 2009). The discriminatory treatment of migrants is time and again justified by political figures on the basis of the social and economic crises facing South Africa where an estimated half of the population is unemployed, even though these claims are by and large unsubstantiated by empirical evidence. As a result, many grueling accounts of violence against foreign migrants have been recorded between 1998 and 2018 ^1^

#### Medical xenophobia in South Africa

State institutions have been severely implicated in both perpetrating and sustaining xenophobia and the violence that is its manifestation. For example, the South African Police Service’s response to the 2008 violence in protecting victims was criticized for being quite ambivalent and leaving a lot to be desired (Polzer and Takabvirwa, 2010). Hence, beyond violence practiced by ‘bodies or actions of poor individuals’ (Mupotsa and Kreutzfeldt, 2016), Neocosmos (2008) notes that xenophobia can also be institutional or structural. Xenophobia is not only a violent phenomenon but it is also manifested in South African practices through the exclusion and discrimination of foreigners in various institutions like banks, hospitals, the Department of Home Affairs, police, and social service providers. It has become somewhat institutionalized in practices of ‘street-level bureaucrats’ (Lipsky, 2010) like immigration officers, health care providers, police officers and policy-makers since 1994 because of the perceived ‘threat’ migrants pose. Through xenophobia, institutions have been used to exclude the ‘other’ through practice and not by design (Adjai and Lazaridis, 2013).

Obviously, not all employees of the state are in a direct position to exploit vulnerable migrants, asylum seekers and refugees for personal gain (Crush and Tawodzera, 2014). While they come into contact with migrants and refugees in the course of their jobs, those in ‘helping professions’ like teachers, social workers and health care providers do not play an active role in South Africa’s enforcement and immigration control machinery. Crush and Tawodzera (2014) argue that they do, however, in the absence of official directives to the contrary (and sometimes despite such directives) have the power to withhold services and can certainly influence the way in which those services are delivered.

However, the ‘single story’ of the exclusionary nature of this discretion seems to have been overstated. Definitions of medical xenophobia presented by scholars like Crush and Tawodzera (2014) and Zihindula et al. (2015) negate the notion that ‘difference’ and ‘outsiders’ are subjectively and socially constructed and negotiated, which is critical to how xenophobic discrimination is experienced (or not) by both locals and migrants. In this sense, these definitions are inconsistent with broader understandings of xenophobia as a phenomenon that transcends nationality, cutting, rather, across different, temporal social constructions of whose body is perceived by the community as an ‘outsider’.

In the course of conducting fieldwork, there was one instance observed where a nurse treated three local school children badly by being particularly rude to them. In an interview, Thelma who was a Clerk at the clinic reduced this incident to being a case of generalized ‘bad attitudes’ drawing on her three years of working in the clinic. This example demonstrates how the predominant simplistic accounts of medical xenophobia easily fall into the trap of framing certain incidences as xenophobia, without a sufficient engagement with mediating factors that may not be directly associated with nationality. Certainly, as illustrated in this instance, ‘bodily’ contingencies like age that dictates the top-down nature of relationships in most African societies could very well have contributed to such treatment. Similarly, the frustrations related to working in understaffed workplaces could also be a contributing factor. Therefore, to describe medical xenophobia as ‘the negative attitudes of health sector professionals and employees towards refugees and migrants on the job’ (Crush and Tawodzera, 2011, p. 1) is broad and could also mean that the nurse in question was being xenophobic, which is in this case an inaccurate conclusion.

In this regard, the notion of medical xenophobia exhibits a lack of theoretical rigor and consistency, which is akin to the ‘multidimensional’ concept of exclusion, which O’Reilly (2005, p. 81) describes as ‘naïvely heuristic and tautological in that it identifies social problems and then labels them as aspects of exclusion’. The notion of medical xenophobia, like the multidimensional concept of exclusion:

…is not guided by any particular social science paradigm or theorization of what either exclusion or inclusion is. Its lack of theoretical rigour, however, means that the absence of a strong ideological orientation allows a relatively open approach to identifying exclusion, even if its symptoms and conditions are not systematically understood (O’Reilly, 2005, p. 81).

There is to some extent an acknowledgment of this weakness by scholars who argue this position. Crush and Tawodzera (2014) for example advance the weakness that they found in previous studies that confirm that migrants face myriad problems when they try to access government health services in South Africa. Crush and Tawodzera (2014) add that these studies draw attention to the magnitude of the problem without generally distinguishing poor treatment that is meted out to all patients in an overburdened and under-resourced public health care system from poor treatment that is a direct consequence of the nationality and origins of the patient. They are careful enough to recognize that a primary challenge in assessing the reasons for the ill-treatment of migrants in South Africa, then, is the assumption—often held by migrants themselves—that when they are denied services or treated badly by health care providers, it is simply because they are foreigners. It is only when the patient is treated badly or denied treatment precisely because they are foreigners, or are denigrated verbally or physically for being non-South African, that there is evidence of medical xenophobia at work (Crush and Tawodzera, 2014).

They present the question that while such treatment is clearly unacceptable and disregards patient rights, is it necessarily xenophobic? In that regard, they suggest that, simply because a migrant from another country receives poor or abusive treatment at a South African health facility, we also cannot automatically assume that it is because they are foreign or that this is evidence of xenophobia. In their interviews in Cape Town and Johannesburg, they uncover many instances of poor treatment of patients that could not be definitively ascribed to the fact that the patient was a ‘foreigner’.

There are, however, a few limitations in Crush and Tawodzera’s analysis. First, while recognizing and acknowledging these nuances, their analysis is limited only to those treatment narratives that can be attributed to medical xenophobia and then they look for common themes and patterns in those narratives. At best, Crush and Tawodzera (2014) hint at the influence that being overloaded with work and being under-resourced has on the conduct of the health care providers, while at the same time ignoring how this influences the conclusions they make.

Second, Crush and Tawodzera (2014) further argue that the most important obstacle for Zimbabwean migrants trying to access public health care in South Africa is the denial of treatment to those who cannot produce the ‘correct’ documentation. The respondents in their study indeed said that it was very difficult for them to get treatment at clinics and hospitals if they did not first produce documents verifying their right to be in South Africa. Others have also made similar conclusions (Zihindula et al., 2015; Crush and Tawodzera, 2014; Hunter-Adams and Rother, 2017; Misago et al., 2010; Human Rights Watch, 2009; Landau, 2007; Pursell; 2007; Nkosi, 2014). Crush and Tawodzera (2014) conclude that, in the public sector, those who cannot produce evidence of their legal right to be in South Africa are regularly refused treatment or turned away from government hospitals and clinics, no matter how sick they are (Crush and Tawodzera, 2014). However, it remains unclear in their analysis what the scale of this challenge is, partly because they do not account for the agency of migrants who navigate the health care system and the positive discretion or agency of health care providers who may act in migrants’ favor to ensure treatment for all. Moreover, the centrality of the ‘body’ as a site for the negotiation of belonging and ‘stateless citizenship’ within the medical system and under what conditions this may occur is not sufficiently invoked.

### Citizenship and public health: Bureaucratic incorporation and therapeutic citizenship

In as much as Section 27 of the South African constitution includes the provision that everyone has a right to health care, national citizenship continues to play a key mediating role in the experiences of individuals that interact with South Africa’s public health care system. However, this often happens in less direct ways than implied in the autochthony debate posited by Nyamnjoh (2006), Geschiere (2009), Landau (2010), Morreira (2010), and Klotz (2016). For example, in what Willen (2012) calls the embodiment of migrant illegality in both the epidemiological and phenomenological sense, negative narratives about the xenophobic way health care providers treat migrants may cause undocumented migrants to avoid care-seeking for fear of arrest or deportation (Quesada et al., 2011) or internalize exclusionary arguments that they are undeserving of health care (Larchanché, 2012). As Morreira found in her work on undocumented Zimbabwean migrants in Cape Town, being *makwerekwere* (a derogatory South African term for foreigners) indeed results in an embodiment of fear (Morreira, 2010). Therefore, as found by Zihindula et al. (2015), it may be true to a certain extent that undocumented migrants adapt their treatment-seeking behaviors because they are afraid to access health care services in most South African health care institutions (psychosocial effect).

Lipsky (2010) suggests that it would be much of a mistake to infer that ethnic, racial or nationality appeals always prevail in affecting discretionary judgments as that they never prevail. For instance, Lipsky (2010) found that white bureaucrats might be more lenient or tolerant with black clients out of fear of being accused of racial biases. He found San Francisco teachers who over-reacted to the potential for biased behavior by awarding black children good grades when in fact they were not learning at an acceptable rate. This suggests that frontline ‘street-level bureaucrats’ may be inclusive in counterintuitive ways, and this inclusion may be more pronounced in contexts, relations and interactions that are known to be typically discriminatory towards certain bodies. Hence, unlike the exclusion-based and autochthonous notion of medical xenophobia, bureaucratic incorporation and therapeutic citizenship are concepts that could allow migration and health scholarship in South Africa to acknowledge the complexity, counterintuitiveness and ambivalence of the ways in which frontline health care providers might treat migrants in the public health care system through various rationales of moral deservingness mediated by the body.

Bureaucratic incorporation is generally the process whereby public service agencies come to see migrants as ‘simply another set of clients, and act to provide services to them much as they would to any other clients’ (Gell-Redman et al., 2014, p. 2). It counters top-down visions of political control (political incorporation) that expect minority groups to ‘receive political rights and power in the electoral sphere before they receive social rights in lower-order bureaucratic institutions’ (Marrow, 2009, p. 757). In reality, bureaucratic responses to minority groups do not only possess technical expertize and autonomy beyond politicians’ control, but also have internal values that influence their behaviors (Marrow, 2009). In bureaucratic incorporation, undocumented migrants assume de facto legitimacy to be part of civic community, based on a conception of local ‘inhabitance’ or residence (e.g., *jus domicili*) rather than birthright, ancestry, or legalistic citizenship (Marrow, 2012, p. 847). Legitimacy to be seen as part of civic community happens in two ways. The one way is shown by Siziba in his work on Zimbabwean migrants in different neighborhoods of Johannesburg, where conceptions of local ‘inhabitance’ may be mediated by identitive aspects such as language, whose value ‘does not inhere in language itself but in how language as an ‘entry fee’ is received by different interlocutors and in different domains under which specific power relations lie’ (Siziba, 2015). Language may act as an ‘entry fee’ through which the ‘enoughness’ and ‘authenticity’ of its speakers can be evaluated (Siziba, 2015), through what has been termed ‘an avenue of incorporation’ (Worby, 2010).

The other way that bureaucratic incorporation occurs is through a discourse of moral deservingness, which centers the body as a site of negotiation. In an ambivalent system that pushes them to deny care through ‘hidden bureaucratic barriers’ (Marrow, 2012, p. 847), an action that may be inconsistent with their values, this kind of bureaucratic incorporation is characterized by instances where health care providers struggle to cope and resist, yet subscribe to an ethos of ‘what is right for the patient’ (Walter and Schillinger, 2004). This aspect of bureaucratic incorporation is closely aligned with therapeutic citizenship, which is ‘a form of stateless citizenship whereby claims are made on a global order on the basis of one’s biomedical condition, and responsibilities worked out in the context of local moral economies’ (Nguyen et al., 2007, p. 142). As a by-product of biomedicalization and a form of ‘biological citizenship’, therapeutic citizenship represents a shift in the way we understand the governance of bodies and space (Young et al., 2019). Therapeutic citizenship as a form of biological citizenship goes a step beyond healthy citizenship as ‘citizens are encouraged to be healthy not only through the physical management of bodies (e.g., exercise), but also by using biotechnologies for diagnosis, treatment and ongoing (or anticipatory) monitoring’ (Young et al., 2019, p. 3). Looking at citizenship within an era and framework of biomedicalization reframes citizenship, which beyond nationality comes to be ‘governed through both rights and responsibilities: the rights to biotechnologies, treatment and care and the responsibility for the health and well-being of oneself and others’ (Young et al., 2019, p. 3). These rights and responsibilities could be framed around universally accepted principles such as those set by the Joint United Nations Programme on HIV/AIDS (UNAIDS) to have 90% of children screened, 90% in treatment and 90% viral suppression by 2020.

Nguyen’s concept of therapeutic citizenship is concerned with describing ‘how subjects are formed through an assemblage of HIV institutions that make up the global AIDS industry’ (Young et al., 2019, p. 3). This citizenship ‘encompasses activism, peer support and counseling techniques, where citizens learn to tell a story and are triaged into treatment regimes in resource-scarce settings as valuable members of emerging HIV communities’ (Young et al., 2019, p. 3). Wilhelm-Solomon (2013, p. 228) found in Uganda that HIV-positive camp residents developed social relationships around their shared biological condition and through therapeutic practices. Wilhelm-Solomon (2013, p. 232) conceives HIV-based social relations as a ‘biosociality’, which ‘arises from belonging and identity based on a biological condition and shared therapeutic and diagnostic practice’. He adds that new HIV-based support groups may use their disease status ‘to refashion national or transnational conceptions of rights, citizenship, and entitlements’.

In the context of this study, therapeutic citizenship is invoked to show how health care providers come to expect migrant patients living with HIV to portray a certain level of therapeutic ‘responsibility’ (Nguyen et al., 2007, p. 142). This expectation is almost a condition that health care providers use to ‘ignore, stretch, bend, and, if need be, break restrictive government policies to provide ‘more-than-routine’ service to newcomer clients’ they deem ‘worthy’ (Marrow, 2009, p. 759). In as much as bureaucratic incorporation as a process of ‘creaming’ (Lipsky, 2010) may exclude others (‘defaulters’ and clinically ‘unstable’ patients) deemed ‘unworthy’ under criterion of ‘responsibility’, it nonetheless animates the possibility for some migrants to appropriate ‘stateless citizenship’ whereby they can make claims on the basis of their biomedical condition.

Klotz (2016, p. 181) criticizes the analyses of scholars (Geschiere, 2009; Landau, 2010; Morreira, 2010; Nyamnjoh, 2006) for ‘making too many assumptions about the nation-state upon which democracy is built, including their prioritization of nation over state’. In light of this debate, both notions of bureaucratic incorporation and therapeutic citizenship contribute to the problematization of the notion of ‘autochthony’ (Geschiere, 2009) that forms the basis of the ‘methodologically nationalist’ notion of medical xenophobia in which political goals are portrayed as aligned with bureaucratic ones. The actions of health care providers may be ‘highly reflective of their professional orientations and goals, not just of government policies’ (Marrow, 2009, p. 758). The two concepts achieve this because they ‘decenter’ belonging from the state and place morally deserving ‘migrant bodies’ as the central site for the negotiation of emerging forms of belonging and ‘stateless citizenship’ in South Africa. When restrictive government policies collide with their beliefs about fairness and appropriate action toward their clients, health care providers may adopt service-oriented professional norms, which magnify their views of themselves as ‘advocates’ oriented to the needs of ‘consumer clients’ rather than ‘the system’ (Maynard-Moody and Musheno, 2003).

### Methodology

This article focuses on the interactions between frontline health care providers with three categories of migrant patients living with HIV and on ART: undocumented migrants; migrants without referral letters (‘self referrals’); and non-native speaking migrants in Musina, South Africa. The term ‘migrant’ is used only to capture nationality (Migration Observatory, 2017). Since the study did not interview migrants per se, other criteria like length of stay could not be used to define migrants, as these variables could not be delimited in this study. The term ‘undocumented’ is used throughout the article to describe individuals who currently lack the documentation required to be in South Africa legally (Vearey, 2012, p. 61).

Fieldwork was conducted in Musina; a town that is part of Vhembe district, which lies on the northern Beitbridge border of the Limpopo province, and is composed of four health sub-Districts^2^(Massyn et al., 2015). The district borders on Botswana, Zimbabwe and Mozambique, which makes it a space of high mobility. Most studies on migration and health in South Africa generally tend to take place in Johannesburg and Cape Town, which means that the dominant generalizations about health care providers’ attitudes to migrants in South Africa may not apply indiscriminately to this border town. Public health care facilities in certain areas of the district face challenges of treating foreign nationals from surrounding border areas, who are a part of the border population but are ‘not included in the headcount, in addition to the local population’ (Massyn et al., 2015, p. 6).

Prior to the study, there was no relationship between the researcher and participants. Building on a study conducted by the Migration and Health Project Southern Africa (maHp) in 2015 and its long-standing engagement in Musina, the researcher capitalized on existing relationships and some degree of established rapport in the research site. The main research question guiding the study was: what are the practices, experiences and perspectives of frontline health care providers in a cross-border public primary health care facility associated with high levels of migration?

Non-probability purposive sampling techniques were used to identify respondents. The study was mainly informed by Lipsky’s (2010) theory of street-level bureaucracy, which places emphasis on the role of frontline discretion in service provision. Since decision making in human service bureaucracies occurs primarily in direct face-to-face encounters between clients and frontline ‘street-level bureaucrats’ (Weiner et al., 2004), operationalizing the research question required an ethnographic approach. Qualitative methods were used, namely in-depth interviews and participant observation.

Throughout the month of November 2016, interviews were conducted in English during working hours (tea breaks, weekends and lunch) with ten health care providers who were mainly data capturers, nurses, administrators, clerks and receptionists at the clinic, until data saturation (Tong et al., 2007). This study cohort was composed of three males and seven females. This small sample means that a pattern or generality among health care providers and the relationship between these particular interlocutors cannot be sufficiently determined. While this kind of focus has implications on the the limited possibility for producing a general critique of the literature on medical xenophobia, the reference made in the article to the larger structural and systemic challenges in South Africa’s health care system, and the need to recognize the dextrous ways in which heath care workers ‘make do’ makes the article’s argument stronger.

Elaborate observations of interactions between migrant patients and frontline health care providers at the reception and in consultation rooms were complemented by observations of one sitting of the Vhembe District Migrant Health Forum and another one of the Vhembe District Cross-border Forum. Both consultative forums involved local and Zimbabwean professional nurses, facility managers, administrators and policy-makers from the Limpopo Department of Health. The main discussions in these spaces tended to focus on creating harmonious systems to coordinate continuity of care for communicable diseases between Musina and Beitbridge. It was during these consultations that cordial relationships with participants were strengthened as participants came to see the researcher as part of their team.

In addition to the experiences of migrants in South Africa’s public health care system documented in extant literature, these discussions brought out narratives about the experiences of migrants who were not interviewed as part of this study. There are some limitations to the claim that there is therapeutic citizenship taking place in Musina that are related to the sample bias that did not allow a contrast between the primary responses of frontline health care providers and the voices and experiences of migrants to be established. More ethnographic research that includes the narratives and experiences of both migrants and health care providers is needed to understand the full nature and extent of this phenomenon.

### Navigating ‘blurred’ migration and health policies

#### ‘We don’t arrest persons for becoming sick’: ‘Local inhabitance’ over legalistic citizenship

In spite of several structural and systemic challenges, frontline health care providers in the studied clinic in Musina generally provided most migrants access to public health care services and ART. However, they faced several bureaucratic difficulties in providing ART, which made them suspicious of the motives of migrants. One bureaucratic difficulty health care providers faced in providing ART to migrants was related to documentation. All of the health care providers interviewed claimed that identity documents helped them to effectively identify patients, tally their headcount and minimize ‘loss to follow-up’. Migrant populations in South Africa have indeed been exposed to significant changes in the country’s immigration legislation and policies over the past two decades (Moyo, 2016). There is no room here to go into the details of South Africa’s immigration policy, suffice it to say the hostile immigration environment epitomizes the two-gate system of the Aliens Control Act (1973), which excludes a significant number of migrants by selecting only ‘highly-skilled’ migrants as desirable (Landau and Segatti, 2008; Vanyoro, 2019). The bulk majority of the ‘low-skilled’ migrant labor force is undocumented.

Health care providers perceived undocumented migrants as problematic patients who ‘abused the system’ by assuming different identities in order to ‘hoard’ treatment. This perception was shown by Cindy who was the Nurse in Charge of ART. Cindy had been working in the clinic for one year. Even though our interview was in English, she spoke Venda and traveled approximately 160 km daily between home and work. Cindy had this to say:

We can find that one person has got two files here or three. The first day when he come, he will call another name and surname, because there is nothing that he can be seen that this person is the same. The next week when he come we will open another file, with another name and surname. It happened here, another man he came, calling another name. That man you see, he is collecting being one but he is collecting by two files [Cindy, Nurse in Charge of ART, female].

In an attempt to deal with the perceived challenge of identification, health care providers in the clinic had come up with a system of using the date of birth of an undocumented migrant patient to identify and keep a record of them. The date of birth came to replace the thirteen-character South African identity document, which health care providers routinely use to open patient files. Sharon, a Sotho-speaking nurse at the clinic who was originally from Louis Trichardt Municipality and had recently joined the clinic in the beginning of that year stated that:

When they come, we don’t send them back. We don’t send them out. We open the file for each and every patient, regardless. We don’t even need the passport or ID number. We open the file, for as long as you know your date of birth. We just open the file and then give you the file number. So we’ll continue using that file number. When you come you just produce the file number then they give you the file. Regardless that you came here legally or not, we just offer the care [Sharon, Nurse, female].

The ambivalence of their work environment came out when a few health care providers argued that this system was susceptible to several abuses. For example, one of them, Janet, mentioned that it was easy for undocumented migrant patients to go to several clinics and ‘hoard’ treatment for illicit purposes or to send back to their home countries that she believed did not have enough ART. Janet was an HIV Coordinator who had been working as a professional nurse in Musina for the past ten years. In other words, Janet argued that these patients could not be effectively monitored, which was a violation of patients’ responsibility for the health and well-being of ‘oneself and others’ espoused under therapeutic citizenship. She had this to say:

It’s just that they are not reliable sometimes. That’s the problem on so many things. Because you don’t know this person if it’s Tapiwa or Simbisai [both common Zimbabwean names]. This one who is sitting here, is having this letter, maybe a book or a transfer letter, and you make a mistake you don’t take that transfer letter. Tomorrow somebody will come back with that and say I’m Tapiwa, I’m Rumbidzai. The identity problem it’s an issue to us, because we are unable to identify them (Janet, HIV Coordinator, female).

Common claims made in the narrative of medical xenophobia argue that xenophobic health care providers use such negative perceptions of undocumented migrants to de facto deny them ART and other health care services. However, the health care providers interviewed at the clinic claimed that their perception that undocumented migrants were problematic did not necessarily translate into them denying them health care services and ART. Musina is a border town that lends itself to a particular sociality, which informed an implicit response to migrants that was characterized by a positive collective staff morale at the clinic. Health care providers worked closely with those from Beitbridge through several consultations therefore they saw migrant patients as ‘just another set of clients’. Several of them used notions of morality, ethics and public service to frame their decision-making. These health care providers understood health care as generally a right for everyone in line with Section 27 of the national constitution. Talent who worked as a temporary Clerk and Yvette and Silindile, both Nurses, invoked these moralized and professionalized discourses as a way of describing why local inhabitance was more important to them than legalistic citizenship or regular migration status in the way they rationed services between locals and migrants:

What I know is that me as a clerk I’ve been taught to not discriminate, you know. If you don’t have a passport, a asylum, or what, it means like you can’t be treated? That’s not how we work here, if you have a document or you don’t have, you get treated [Talent, Clerk, male].

Remember the mission of the government or the objective is to assist anybody irrespective of the country, of the race, of the nationality. So we are still sticking on that. We don’t deny anyone a service because he does not have an ID [Yvette, Nurse, female].

It’s not our responsibility as health workers to discriminate whether you come from this country, whether you’ve done this. Our pledge as nurses says, ‘you don’t discriminate according to religion, whatever’. We are supposed to accommodate [Silindile, Nurse, female].

Translated into practice, these sentiments were backed up by several observed cases where undocumented migrants were provided access to health care by the very same health care providers who had alluded that undocumented migrants were problematic patients. For example, these excerpts from the field notes help to corroborate the responses of health care providers with their actual behavior:

Another foreign patient presents herself. The clerk [Thelma] asks her

Clerk: Une pasipoti? [Do you have a passport?]

Patient: Nhasi handina kumbouya nayo ini [Today I did not bring it with me]

The clerk registers her nonetheless (Field notes, Musina, 27-10-2016).

At this point I have been here for 6 hours and no one has been turned away for documentation, address, phone numbers or ill-treated or abused (Field notes, Musina, 27-10-2016).

There was no observed evidence to support the hypothesis that legal status was a deterrent factor among migrants who sought treatment at the public health care clinic in Musina. The situation in Musina was not as simple this hypothesis suggests. There was an ambivalent response to systemic challenges in which health care providers created often unsubstantiated stereotypes of problematic migrant patients while simultaneously coming to see them as simply another set of clients, and acting to ‘provide services to them much as they would to any other clients’ (Gell-Redman et al., 2014, p. 2).

#### Addressing ‘self-referrals’

Frontline health care providers faced bureaucratic difficulty providing ART to migrant patients without referral letters. Health care providers at the consultations were concerned with the clinical importance of referral letters in monitoring and fostering continuity of care among migrant patients living with HIV. They reiterated their distrust of migrant patients living with HIV who presented themselves to the clinic without the necessary referral letters. Ostensibly, the lack of standardized guidelines and systems of patient referral to harmonize and better coordinate HIV treatment in the SADC region resulted in ‘self referrals’: a practice described by health care providers as being adopted by migrant patients living with HIV who came without any referral letter from their initiating facility.

There were several reasons why health care providers needed referral letters. Besides their administrative function, they used referral letters to confirm the HIV status of migrant patients. Yvette even went as far as arguing that migrant patients often lied about their HIV status so as to access HIV treatment for *nyaope*^3^or *whoonga*. She said:

Sometimes you know that some people use the ARVs as drugs you see. So that thing of just giving them by saying that, without evidence, by saying that I’m on treatment. We don’t just give like passing that ‘I have got my treatment’. No! [Yvette, Nurse, female].

Literature suggests that such claims are not substantiated by empirical evidence. So far, studies have identified the robbing of HIV patients’ drugs as the chief way that they get sold for *nyaope* (Chinouya et al., 2017). In a Durban study, Grelotti et al. (2014) found that ARVs were being diverted because some individuals used them recreationally. Media reports in 2008 described how some South African HIV patients and school children smoked ARVs for their ‘hallucinogenic and relaxing effect’ and in 2010, issued numerous reports on whoonga—a cocktail of drugs believed to contain ARVs (Grelotti et al., 2014). The only other reports of recreational ARV use they mention are from the United States where HIV patients were found to abuse and/or divert drugs, and informants described how protease inhibitors, most notably ritonavir, enhanced or prolonged the effects of ecstasy and methamphetamine and were being sold with illicit drugs as ‘cocktails’ (Grelotti et al., 2014; Larkan et al., 2010).

In spite of these unsubstantiated biased assumptions by Yvette, the clerks at the clinic Talent and Thelma maintained that that they registered these ‘suspect’ ‘self referral’ migrant patients nonetheless. Thelma for example argued that:

That referral letter is what is needed actually. They’ll show me *uri* [that] ‘I came with this referral letter from where where where where’. ‘Eh, no problem’, I open a file for him. I direct him straight to the nurse, at the chronic side there. And once they get in they get treated. Those who don’t have [a referral letter] I still refer him or her to the nurse and I don’t know what nurse is saying to him [Thelma, Clerk, female].

Thelma’s response suggests that, while clerks had authority to register ‘self referral’ migrant patients, the decision to administer ART to these migrant patients or not was ultimately at the nurses’ full discretion. This state of affairs further complicates the simplistic notion that frontline health care providers in South Africa turn away migrant patients without necessary documentation. This statement does not sufficiently engage with the role of facility-level hierarchies and individual discretion in the rationing of health care services.

Beyond the role of facility-level hierarchies and individual discretion, the decision to provide ART and access to health care services to ‘self-referral’ migrant patients was also mediated by the ways in which members of ‘emerging HIV communities’ professed their ‘belonging’ through ‘alternative’ forms of knowledge and expertize. For example, Janet claimed that she only provided ART if ‘self-referral’ migrant patients professed knowledge of their medication or brought the container with them to the clinic since there was no practice of repeating HIV tests, checking CD4 counts or viral loads as those whose HIV status could not be ascertained had to be referred to the nearest hospital for retesting:

We can’t deny you treatment if you come in here and said ‘I’m on this treatment, I take it on this time’, ‘I’ve lost my pill’ or ‘I’ve forgoten my pill’ or ‘they have finished’. We can assist you. But if you don’t have any knowledge, it’s a problem [Janet, Nurse, female].

Here, knowledge of the medication one was taking held a key to a particular dimension of ‘biomedicalized’ citizenship which depended on the ‘self-referral’ migrant patient’s ability to ‘confess’ which drug they were taking. ‘Ignorance’ was shunned and could lead to ‘therapeutic ostracization’. By confessing the kind of medication one was taking, health care providers allowed ‘self referral’ migrant patients to appropriate ‘stateless citizenship’ whereby they could make claims on the basis of their biomedical condition, and more importantly by portraying to the health care providers a certain level of therapeutic ‘responsibility’ (Nguyen et al., 2007, p. 142).

### Negotiating language: Lingua franca and informal interpreters

Since there were no positions for official on-site interpreters at the clinic in Musina, health care providers, who mostly spoke Tsonga, Sotho, Shangaan and Venda, reported difficulties in interacting with migrant patients who spoke Swahili, French, Portuguese and Chichewa. Hence, there were several instances observed when bilingual employees who were not formally trained or paid for interpretation served as informal interpreters. There were also times when some health care providers requested patients or family members to translate. Talent described an encounter he had recently had at the clinic reception:

Last time I remember I was treating this other guy with his wife and two children. They don’t know how to speak English, they speak Swahili those people and French. But we were able to treat them. Fortunately there was a soldier guy sitting there on the queue. South African soldier guy. He said, ‘yeah, I think, I can see you are having a tough time there, let me help you, what’s the problem huh?’. They started to talk. He was translating when I was doing everything there [Talent, Clerk, male].

Literature on medical xenophobia does not outline such ways in which staff and local patients can work together to ensure that ‘non-native’ speaking migrant patients access health care services. All the health care providers at the clinic made the effort to connect with migrant patients through informal interpreters, often in extremely demanding circumstances. Because of the close geographical and cultural proximity between Musina and Zimbabwe, which has been observed by others (Dube, 2017; Siziba, 2015), half of the health care providers had learnt sufficient ‘medical Shona’—a majority language spoken in Zimbabwe. Thelma claimed that this was made easier by the similarity between some Venda and Shona words:

I speak Tsonga. It’s not much difficult because I can speak Venda, I can speak Sotho, I can speak all languages from here Musina. Even our neighbour country Shona I can tell touch ups. It’s not different. They link, Venda and Shona. Yah, it’s not much difficult (Thelma, Clerk, female).

*This adoption of ‘medical Shona’ was also observed*.

One foreign patient does not have a phone number. The male clerk [Talent] is speaking in Venda but at this point he seems to have adapted to Shona. He addresses her in Shona, with a tone of concern, ‘Kana muchodzoka next time mudzoke nenumber handiti?’ [‘When you come back next time please bring your phone number, okay?’]. She nods in approval. He allows her to proceed for her vital signs [Field notes, Musina, 3-11-2016].

Despite these innovations, there was still need to meet the needs of half the monolingual health care providers, and to facilitate quality access to care for those whose languages were not so easily accessible to them. There were no positions for official on-site interpreters and no in-service training programs for interpreting techniques. Therefore, while bilingual employees were not formally trained or paid for interpretation, they frequently served as informal interpreters. Cindy’s experiences in providing health care to migrant patients in Musina who often spoke a different language from hers sums up the challenges of monolinguism succinctly. She said:

The nurses who are originally from here in Musina, they can speak their languages because maybe most of them they are originally from there. I don’t know. But maybe it’s because they’ve been staying with them for a long time but when it comes to other communication problems those yah. Like myself, when I came last year, it was a problem because I can only speak Zulu, Tswana, Venda. At least I can speak Tsonga, but when it comes to Shona and Chichewa it was a problem (Cindy, Nurse in Charge of ART, female).

The use of informal interpreters was a widespread practice and the role was highly fluid and invoked as a matter of situational convenience. Who got to be an interpreter was not limited to health care providers, as in some situations, the nurses made use of their own discretion and called upon the services of other non-medical employees, patients, family members fluent in the language in question. Janet described her dependence on a member of the cleaning staff for interpretation:

We don’t have [interpreter services]. So we rely, like which language is it, which language? I’ve forgoten but there’s this difficult language in Africa ^4^We have got somebody who is a cleaner who understands that. So which means we will have to call them (Janet, Nurse, female).

It goes without saying that for conditions like HIV, resorting to these practices could have negative consequences on issues of confidentiality. Nonetheless, the phenomenon of adopting lingua franca and informal interpreters also adds to the complexity of the notion of medical xenophobia, which inherently presumes a kind of ‘rational’ nationalistic response to language difficulty in which health care providers de facto use language as a vehicle of discrimination (Hunter-Adams and Rother, 2017).

## Concluding remarks

This study found insufficient evidence to suggest that health care providers in Musina were indiscriminately using language, documentation and referral letters as vehicles to discriminate against migrant patients. This position is more consistent with existing studies in South Africa (Vearey et al., 2016; Pophiwa, 2010; Makandwa, 2014) than others (Zihindula et al., 2015; Crush and Tawodzera, 2014; Hunter-Adams and Rother, 2017; Misago et al., 2010; Human Rights Watch, 2009; Landau, 2007; Pursell, 2007; Nkosi, 2014). Both notions of bureaucratic incorporation and therapeutic citizenship contribute to the problematization of the notion of ‘autochthony’ (Geschiere, 2009) that forms the basis of the notion of medical xenophobia, by hinting at the centrality of morally deserving ‘migrant bodies’ (not nation or state) as sites for the negotiation of emerging forms of belonging and ‘stateless citizenship’ in South Africa. Beyond national citizenship and legal status, the study unpacked emerging processes of bureaucratic incorporation (Marrow, 2012; Gell-Redman et al., 2014; Walter and Schillinger, 2004) and to some extent therapeutic citizenship (Young et al., 2019; Nguyen et al., 2007; Wilhelm-Solomon, 2013). These processes counter top-down visions of political control (political incorporation) that expect minority groups to ‘receive political rights and power in the electoral sphere before they receive social rights in lower-order bureaucratic institutions’ (Marrow, 2009, p. 757).

Bureaucratic incorporation took place in an ambivalent public health care system that pushes health care providers to deny ‘indigent’ migrants care through ‘hidden bureaucratic barriers’. It appears that this exclusionary action was inconsistent with some of their values (Marrow, 2012, p. 847). Health care providers thus struggled to cope and resist, yet subscribed to an ethos of ‘what is right for the patient’ (Marrow, 2012). Therapeutic citizenship, to a lesser extent, mediated the appropriation of ‘stateless citizenship’ whereby health care providers allowed ‘self referral’ migrant patients to make claims on the basis of their biomedical condition by portraying to them a certain level of therapeutic ‘responsibility’. This is different from the way that others have deployed this concept elsewhere as it draws on the perceptions and experiences of health care providers and not patients themselves in order to show the way health care providers’ bodily expectations influence the way they respond to the needs of migrants.

It is this article’s argument that medical xenophobia does not adequately capture the full extent of complexity, ambivalence and range of possible experiences of non-nationals in South Africa’s public health care system. Innovation, creativity and compromise all helped health care providers in Musina to bypass health systems that do not consider the precarity of migrant patients. So far, this agency in the dominant migration and health literature in Southern Africa has been assumed to be about health care providers’ ways of working that negatively affect migrants rather than exploring ways of helping them.

Going forward, research on interactions between health care providers and migrant patient needs to be explored in a manner that also considers the South African public health system’s constraints and the centrality of discretion. Multiple forms of othering, and how they are mediated, need an approach that goes beyond methodological nationalism by conceptualizing nationality, citizenship and legal status as their unit of analysis. Simply alleging that health care providers are xenophobic only provides a limited analysis. In terms of possible interventions, South Africa’s policy-makers need to recognize the importance of informal elements such as human relationships, communication networks, leadership and motivation in strengthening function of the country’s ailing public health care system. The informal practices of health care providers in addressing challenges related to documentation, referrals and language need to be strengthened. Moreover, broadly speaking, the need for less ‘blurred’ policy responses to communicable diseases in South (ern) Africa that adequately engage with mobility and harmonized referral systems remains an urgent concern.

## Footnotes

1 **Notes** Xenophobic violence has become a perennial feature in post-apartheid South Africa (Mlilo and Misago, 2019). Many grueling accounts of violence against foreign migrants have been recorded between 1998 and 2008 (Crush and Frayne, 2010). One of the most notorious occurrences of xenophobic violence in the world was the May 2008 violence (Vanyoro and Ncube, 2018). Most scholars agree that 62 people lost their lives, a third of whom were local inhabitants, whereas at least 670 were wounded; dozens raped and more than 100,000 displaced. Mozambican Ernesto Alfabeto Nhamuave, who was 35-years-old, was beaten, stabbed and set alight in Ramaphosa informal settlement on the East Rand (SA History, 17 April, 2015). There was another recurrence of xenophobia in 2015, starting off in the township of Soweto and manifesting more acutely in Durban and across other parts of Gauteng (Vanyoro and Ncube, 2018).

2 This fieldwork was part of a larger research project conducted in both Musina and Johannesburg.

3 Nyaope is a uniquely South African street drug that is highly addictive and destructive. It is a fine white powder that is usually combined with marijuana (dagga) and smoked.

4 Once it occurred to her, Janet later told me after the interview that when she spoke about ‘this difficult language in Africa’ she was actually referring to Swahili.
